# Experimental dataset on adsorption of Arsenic from aqueous solution using Chitosan extracted from shrimp waste; optimization by response surface methodology with central composite design

**DOI:** 10.1016/j.dib.2018.09.003

**Published:** 2018-09-07

**Authors:** Mohammad Hadi Dehghani, Mohammad Maroosi, Zoha Heidarinejad

**Affiliations:** a Department of Environmental Health Engineering, School of Public Health, Tehran University of Medical Sciences, Tehran, Iran; b Institute for Environmental Research, Center for Solid Waste Research, Tehran University of Medical Sciences, Tehran, Iran; c Department of Environmental Health Engineering, Faculty of Health, Hormozgan University of Medical Sciences, Bandar Abbas, Iran

**Keywords:** Chitosan, Arsenic, Aqueous solution, Adsorption

## Abstract

The aim of data was to evaluate the efficiency of chitosan extracted from shrimp waste for Arsenic adsorption and optimization by response surface methodology (RSM) with central composite design (CCD). The data showed that, with increasing contact time, the amount of adsorption increased and the optimal contact time was about 60 min. With increasing pH decreased adsorption, although this reduction was not significant. The optimum pH was obtained at 4.41. The average amount of adsorbent capacity was also about 1.3 mg/g. Overall, chitosan extracted from shrimp waste could be considered as an efficient material for the adsorption of Arsenic from aqueous solution.

## Specifications table

**Table:** 

Subject area	Environmental Science
More specific subject area	Adsorption
Type of data	Tables, Figures
How data was acquired	– The tests of As(V) adsorption with chitosan were performed under different initial As(V) concentrations, initial pH levels, contact time and chitosan dosages. – The response surface methodology (RSM) was used to optimize the factors affecting the adsorption and interaction of them, and the central composite design (CCD) was used to determine the number of tests. – The concentration of As(V) was measured with an inductively coupled plasma-mass spectrograph.
Data format	Analyzed
Experimental factors	– The chitosan was prepared from the waste of shrimp waste. – Data of chitosan were acquired for As(V) removal from aqueous solution.
Experimental features	chitosan for As (V) adsorption from aqueous solution
Data source location	Tehran University of Medical Sciences, Tehran, Iran.
Data accessibility	Data are available in article
Related research article	Dobaradaran S, Nabipour I, Mahvi AH, Keshtkar M, Elmi F, Amanollahzade F, et al. Fluoride removal from aqueous solutions using shrimp shell waste as a cheap biosorbent. Fluoride. 2014;47(3):253–7 [6]

## Value of the data

• The data showed that chitosan extracted from shrimp waste can be used as an inexpensive adsorbent for arsenic removal of water and wastewater.
• This data offers a simple method for preparation of adsorbent from shrimp waste.
• This data article presents a user friendly-statistical method (RSM) to optimize Arsenic ions removal from water and wastewater using adsorption process.
• This dataset will be beneficial for researchers who want to achieve good As(V) adsorption capacities with chitosan extracted from shrimp waste and Arsenic ions removal from industrial wastewaters.

## 1 Data

**Fig. 1:**
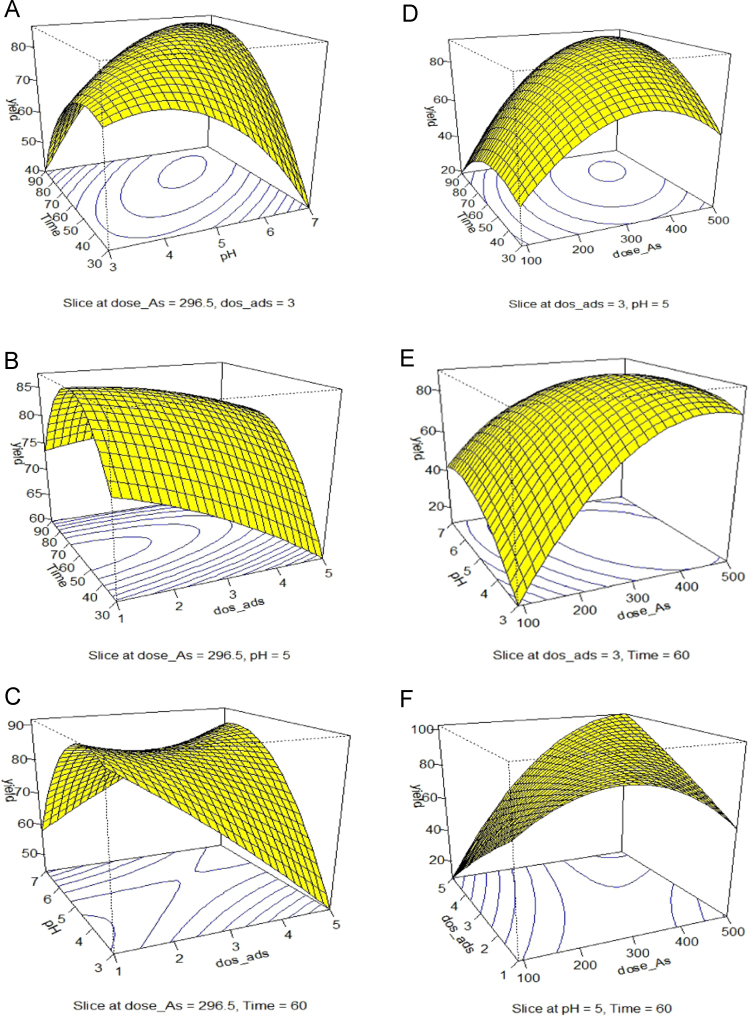
Central composite design 3-D surface plots showing effect of (1A) pH and contact time, (1B) contact time and adsorbent dosage, (1C) adsorbent dosage and pH, (1D) contact time and arsenic concentration, (1E) arsenic concentration and pH, (1F) arsenic concentration and adsorbent dosage, on Arsenic removal efficiency with the adsorbent.

**Fig. 2:**
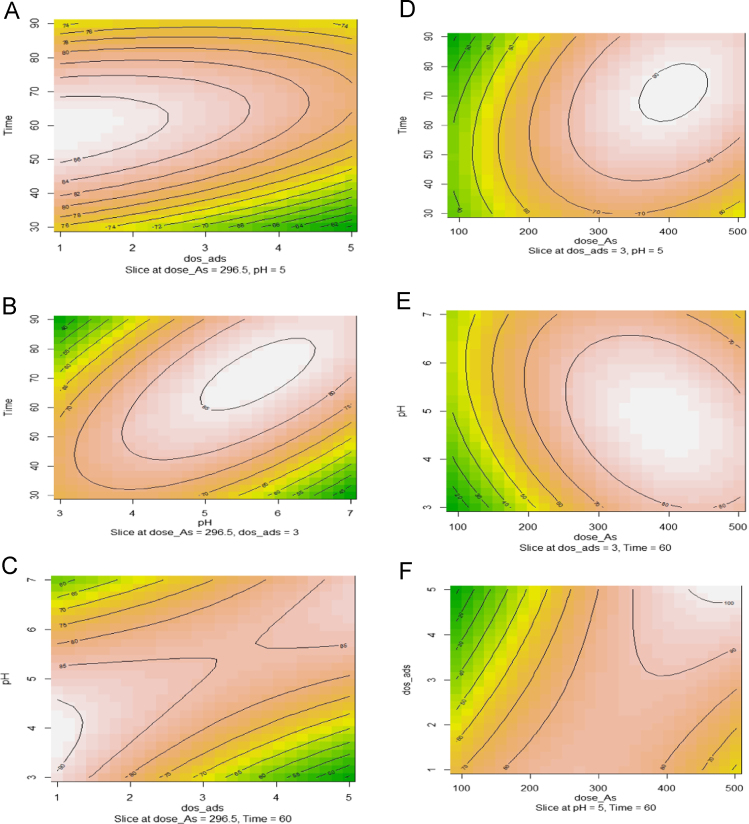
Contour plots for the interaction effect of variables on the Arsenic removal. (2A) contact time and adsorbent dose (g/L). (2B) contact time and pH. (2C) pH and adsorbent dosage (2D) contact time and arsenic concentration, (2E) pH and arsenic concentration (2F) adsorbent dose (g/L) and arsenic concentration (mg/L).

Experimental versus predicted adsorption efficiencies for arsenic removal are also illustrated in Tables 1 and 2. Variables constraints and predicted removal of optimization of arsenic adsorption by Chitosan presented in Table 3. Analysis of variance (ANOVA) for the fitted polynomial model for Arsenic adsorption by Chitosan reported in Table 4. Fig. 1(A–F) shows the Central composite design 3-D surface plots of the interaction effects of pH, contact time, arsenic concentration and adsorbent dosage chitosan extracted from shrimp waste on arsenic removal. The contour plots for the interaction effect of variables on the arsenic removal shows in Fig. 2A–F. Data on analyses showed that the data follow a Second-degree reaction. The data of the Pearson coefficient resulted from ANOVA showed that the contact time (3.58 × 10^− 16^) and the adsorbent dosage (3.16 × 10^− 16^) had a greater effect on the adsorption reaction.

**Table 1:** Center indexes and dispersion indexes of arsenic removal with chitosan.

Mean	26.02204
Standard Error	0.661758
Median	26.47059
Standard Deviation	3.173682
Sample Variance	10.07226

**Table 2:** Design of test factors using R software.

	dose_As	x1	dos_ads	x2	pH	x3	Time	x4	Block
1	194	− 1	2	− 1	4	− 1	45	− 1	1
2	399	1	2	− 1	4	− 1	45	− 1	1
3	194	− 1	4	1	4	− 1	45	− 1	1
4	399	1	4	1	4	− 1	45	− 1	1
5	194	− 1	2	− 1	6	1	45	− 1	1
6	399	1	2	− 1	6	1	45	− 1	1
7	194	− 1	4	1	6	1	45	− 1	1
8	399	1	4	1	6	1	45	− 1	1
9	194	− 1	2	− 1	4	− 1	75	1	1
10	399	1	2	− 1	4	− 1	75	1	1
11	194	− 1	4	1	4	− 1	75	1	1
12	399	1	4	1	4	− 1	75	1	1
13	194	− 1	2	− 1	6	1	75	1	1
14	399	1	2	− 1	6	1	75	1	1
15	194	− 1	4	1	6	1	75	1	1
16	399	1	4	1	6	1	75	1	1
17	296.5	0	3	0	5	0	60	0	1
18	296.5	0	3	0	5	0	60	0	1
19	91.5	− 2	3	0	5	0	60	0	2
20	501.5	2	3	0	5	0	60	0	2
21	296.5	0	1	− 2	5	0	60	0	2
22	296.5	0	5	2	5	0	60	0	2
23	296.5	0	3	0	3	− 2	60	0	2
24	296.5	0	3	0	7	2	60	0	2
25	296.5	0	3	0	5	0	30	− 2	2
26	296.5	0	3	0	5	0	90	2	2
27	296.5	0	3	0	5	0	60	0	2
28	296.5	0	3	0	5	0	60	0	2
29	296.5	0	3	0	5	0	60	0	2
30	296.5	0	3	0	5	0	60	0	2
31	296.5	0	3	0	5	0	60	0	2
32	296.5	0	3	0	5	0	60	0	2

**Table 3:** The results obtained from the removal of arsenic by chitosan.

Run.order	dose_As	dos_ads	pH	time	Result	removed	%removal
1	306	3	5	60	231	75	24.51
2	397	4	6	75	274	123	30.98
3	397	4	6	45	318	79	19.90
4	397	2	4	75	298	99	24.94
5	194	4	6	75	126	68	35.05
6	306	3	5	60	221	85	27.78
7	306	3	5	60	225	81	26.47
8	306	3	5	60	223	83	27.12
9	194	4	4	75	121	73	37.63
10	397	4	4	45	314	83	20.91
11	194	4	6	45	154	40	20.62
12	399	2	6	75	301	98	24.56
13	306	3	5	60	227	79	25.82
14	194	2	6	75	131	63	32.47
15	194	2	4	45	152	42	21.65
16	397	4	4	75	272	125	31.49
17	397	2	4	45	325	72	18.14
18	306	3	5	60	224	82	26.80
19	194	4	4	45	151	43	22.16
20	397	2	6	45	339	58	14.61
21	306	3	5	60	222	84	27.45
22	194	2	4	75	133	61	31.44
23	194	2	6	45	153	41	21.13
24	306	3	5	60	226	80	26.14
25	306	3	5	30	276	30	9.80
26	306	3	5	90	198	93	35.29
27	306	3	5	60	223	83	27.12
28	306	3	5	60	225	81	26.47
29	306	3	5	60	224	82	26.80
30	306	3	5	60	221	85	27.78
31	306	3	5	60	222	84	27.45
32	306	3	5	60	223	83	27.12

**Table 4:** One-way ANOVA to determine the effective factors on the reaction of Arsenic adsorption with chitosan.

ANOVA					
	Df	Sum Sq	Mean Sq	F value	Pr(> *F*)
dose_As	1	6026	6026	770.325	3.16E-16***
dos_ads	1	630	630	80.505	4.61E-08***
pH	1	50	50	6.435	0.020661*
Time	1	5942	5942	759.515	3.58E-16***
I(dose_As^2^)	1	156	156	19.995	0.000295***
I(dos_ads^2^)	1	6	6	0.733	0.403036
I(Time^2^)	1	730	730	93.359	1.51E-08***
dose_As:dos_ads	1	275	275	35.17	1.30E-05***
dose_As:pH	1	21	21	2.704	0.117451
dose_As:Time	1	165	165	21.078	0.000227***
dos_ads:pH	1	0	0	0	0.982592
dos_ads:Time	1	92	92	11.796	0.002956**
pH:Time	1	8	8	1.081	0.312307
Residuals	18	141	8

## 2 Experimental design, materials and methods

### 2.1 Materials

All chemicals used in this data article such as Na_2_HAsO_4_·7H_2_O, HCl, and NaOH were analytical grade. Arsenic from a 1000 ppm stock solution of sodium arsenate 7-hydrate was used to prepare solutions of the required arsenic concentration [1], [2], [3], [4], [5], [6].

### 2.2 Preparation of chitosan

Chitosan was used in the laboratory to extract shrimp waste. Shrimp waste was crushed after drying by the household grinder. Then demineralized in 1 N HCl is added at a ratio of 1 to 20 for 2 h with around 125 rpm was stirred [6], [7]. The acid mixture of shrimp waste was placed at room temperature for 24 h to remove its minerals, including calcium carbonate. The solution was then filtered with Whatman filter paper and dried at room temperature for 24 h [6], [8], [9], [10]. The powder obtained with a weight ratio of 1 to 20 was mixed with 1 N sodium hydroxide and placed on a stirrer at 60 rpm for 4 h to extract the chitin. Then, the obtained Chitin was mixed in sodium hydroxide 50% w/w for 2 h and placed on a stirrer at 100 °C. Finally, the obtained materials were chitosan and used for arsenic adsorption experiments [6], [8], [9], [10], [11], [12].

### 2.3 Design of experiments

The entire batch adsorption experiments were carried out in 50 mL Erlenmeyer flask. The pH of the solutions were adjusted prior to the adsorption by using 0.1 M solutions of HCl and NaOH (16–19). The effects of operational parameters including pH (4, 5 and 6), contact time (30, 45, 60, 75 and 90 min), initial As (V) concentration (200, 300 and 400 mg/L), and adsorbent dosage (2, 3 and 4 mg/L) were assessed. The samples were stirred at 250 rpm for given contact times and after centrifugation at 2000 rpm, and passing through a 0.2 μm membrane filter, Then, the concentration of Arsenic was determined by ICP device [2], [7]. The design of experiments was carried out using central composite design [9]. Then use the data and its analysis in software R (version R 3.5.1) to response surface methodology (RSM) factors affecting the optimum value was determined [1], [3], [5]. The complete design of the factor was made for four independent variables in three levels with 4 center points and 2 axial points. Experiments were performed in 2 blocks and repeated twice.
